# Supplementary material for: Membrane Mechanics of Endocytosis in Cells with Turgor
Source: PLoS Comput Biol. 2015 Oct 30;11(10):e1004538. doi: 10.1371/journal.pcbi.1004538 (PMC4627814; doi:10.1371/journal.pcbi.1004538)
Supplement: S1 Text — 1. Membrane shape. 2. Forces. 3. Shape instability (PDF) [file pcbi.1004538.s001.pdf]

Membrane Mechanics of Endocytosis in Cells with Turgor
Supplementary information

Serge Dmitrieff and François Nédélec*

Cell Biology and Biophysics Unit, European Molecular Biology laboratory,
Meyerhofstrasse 1, 69123 Heidelberg, Germany.

* Corresponding author : nedelec@embl.de

1 Membrane shape

1.1 Differential equations

The membrane shape equations correspond to the minimum of the membrane energy (main text, equation 1). To derive these equations, we renormalize energies by κ and distances by R_Π, without loss of generality. The typical scale of forces will be $f_\Pi = 2\pi\kappa/R_\Pi$. Following earlier work [1], we assume rotational symmetry around the vertical axis, which is defined as the direction normal to the cell wall, orientated towards the cell inside. Let Z be the distance from the cell wall along this axis, R the distance from the symmetry axis, s the arclength along the membrane, and ψ the angle of the membrane with the horizontal axis (see Fig. 1, left), all being dimensionless quantities. Additionally, we define $\bar{\sigma} = {R_\Pi}^2\sigma/\kappa$, $f = f_a/f_\Pi$, $\bar{C}_0 = R_\Pi C_0$ and $\bar{\Pi} = 1/2$. To impose the geometric relations $\partial R/\partial s = \dot{R} = \cos(\psi)$ and $\dot{Z} = -\sin(\psi)$, we use two Lagrange multipliers ν and η. The renormalized energy $\mathcal{F}'$ finally reads :

$$\mathcal{F}' = 2\pi \int_S \mathcal{L}(R, \psi, \dot{\psi}) ds \,, \tag{1}$$

with $\mathcal{L}$ the renormalized energy density:

$$\mathcal{L} = \frac{R}{2}\left(\dot{\psi} + \frac{\sin\psi}{R} - \bar{C_0}\right)^2 + \bar{\sigma} R + \frac{\bar{\Pi} R^2}{2}\sin\psi - f\sin\psi + \nu(\dot{R} - \cos\psi) + \eta(\dot{Z} + \sin\psi)\,, \quad (2)$$

where the dot indicate the derivative with respect to the arc length s. From these, we derive the Euler-Lagrange equations [1] :

$$\ddot{\psi} = \frac{\cos\psi}{R}\left(\frac{\sin\psi}{R} - \dot{\psi} + \eta - f\right) + \frac{\bar{\Pi}}{2} R\cos\psi + \frac{\nu}{R}\sin\psi\,, \quad (3)$$

$$\dot{\nu} = \frac{1}{2}(\dot{\psi} - \bar{C_0})^2 - \frac{\sin^2\psi}{2R^2} + \bar{\sigma} + \bar{\Pi} R\sin\psi\,, \quad (4)$$

$$\dot{\eta} = 0\,, \quad (5)$$

which are valid between $R = 0$ (the center of the invagination) and $R = R_i$, after which the membrane is in contact with the cell wall. Since the energy involves $\dot{\psi}$, ψ has to be continuous and hence $\psi = 0$ at $R = R_i$, whereas $\dot{\psi}$ can be discontinuous. Canceling the boundary term $[\nu R_i]$ imposes $\nu(0) = 0$ (as R_i is a free parameter) and canceling the Hamiltonian [1] imposes $\dot{\psi}(0) = -\sqrt{\bar{C_0}^2 + 2\bar{\sigma}}$. For simplicity, we assumed $\bar{C_0}$ to be constant over the whole membrane surface, an approximation discussed later.

While assuming axisymmetry may seem a strong approximation, minimizing Helfrich's Hamiltonian will produce axisymmetric shapes, and a more general theory would yield the same result, unless the force f_a is not orthogonal to the cell wall, or if the membrane properties are severely anisotropic [2]. Experimentally, we see membrane profiles that are not perfectly axisymmetric [3], which is expected considering the small size of the actin machinery, where possibly as few as 10 filaments are polymerising at any time [4].

1.2 Membrane with anisotropic coat

To describe a membrane coated with anisotropic coat proteins such as Rvs167, we added a term favoring a radius R_0 to the energy density [5] :

$$\mathcal{L}' = \mathcal{L} + \frac{\bar{\Gamma}}{2} R\left(\frac{1}{R} - \frac{1}{\bar{R_0}}\right)^2\,, \quad (6)$$

in which $\mathcal{L}'$ is the new renormalized energy density, $\bar{\Gamma} = \Gamma/\kappa$ and $\bar{R_0} = R_0/R_\Pi$.

The differential equation for $\ddot{\psi}$ is unchanged (equation 3) but a term $\frac{1}{2}\bar{\Gamma}(1/R - 1/\bar{R}_0)^2$ is added to $\dot{\nu}$ (equation 4). Additionally, the boundary value of $\dot{\psi}$ is now :

$$\dot{\psi}(0) = -\sqrt{\bar{C_0}^2 + 2\bar{\sigma} + \bar{\Gamma}\frac{(R - \bar{R}_0)^2}{R^2 \bar{R_0}^2}} \tag{7}$$

1.3 Numerical solutions

To find the membrane shape, we integrated equations 3-5 from $R = R_i$ down to $R \to 0$, i.e. taking a negative ds. Because of the divergence at $R = 0$, in practice we integrated from R_i to a non-zero cutoff $\epsilon = 0.004$, that was chosen to be smaller than a protein. We used a shooting method [6] to find the values of R_i and f such that the membrane reaches $Z = L$ and $\psi = 0$ at $R = \epsilon$. Because of the strong non-linearity in the equations, most initial values of R_i and f will lead to a diverging shape. However, by scanning randomly for initial values of R_i and f for a given L, we could find parameters that gave a physical solution to the equations.

To compute membrane shape for a range of L for a given value of $\bar{C_0}$ and $\bar{\sigma}$, we started from a small value of L and determined the best $\{R_i, f\}$ by a random search coupled to a shooting method. Then, we looked for the solution for $L + \delta L$ using the shooting method starting from the same R_i and f. When δL was small (typically 10^{-6}—$10^{-3} R_\Pi$), the shooting method usually converged, unless a bifurcation was reached. To find solutions for other parameters $\bar{C_0}$ and $\bar{\sigma}$, we either started another random parameter search coupled to the shooting method, or started from a known solution, and slowly changed the $\bar{C_0}$ and/or $\bar{\sigma}$ (again with small steps).

1.4 Heterogeneous membrane

To model the existence of membrane heterogeneities, namely the clathrin-rich tip of the invagination, we introduced a non-constant rigidity $\kappa \mapsto \kappa\,\alpha(s)$. Assuming $\alpha(s)$ to be

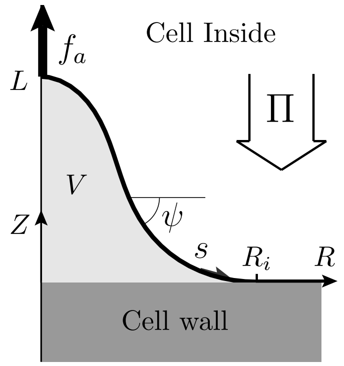

Figure 1. Diagram of the endocytic process and its parametrization. We assumed rotational symmetry around the Z axis, normal to the cell wall. The shape is parametrized by the curvilinear abscissa s, the distance R from the symmetry axis, and the angle ψ. L is the maximal height of the invagination, R_i is the distance beyond which the membrane is in contact with the cell wall, and Π is the difference of hydrostatic pressure across the plasma membrane.

derivable, this changes the differential equations for $\ddot{\psi}$:

$$\ddot{\psi} = \frac{\cos\psi}{R}\left(\frac{\sin\psi}{R} - \dot{\psi} + \frac{\eta - f}{\alpha}\right) + \frac{\bar{\Pi}}{2\alpha}R\cos\psi + \frac{\nu}{\alpha R}\sin\psi - \frac{\dot{\alpha}}{\alpha}\left(\dot{\psi} + \frac{\sin\psi}{R} - C_0\right) \quad (8)$$

$$\dot{\nu} = \frac{\alpha}{2}(\dot{\psi} - \bar{C_0})^2 - \alpha\frac{\sin^2\psi}{2R^2} + \bar{\sigma} + \bar{\Pi}R\sin\psi \quad (9)$$

$$\dot{\eta} = 0\,. \quad (10)$$

For $\alpha(s)$ we used a function varying smoothly between 1 and α_{min}, over a width Δs :

$$\alpha(s) = 1 \qquad \text{if } s \leq s_1 \quad (11)$$

$$\alpha(s) = 1 + (\alpha_{min} - 1)\left(1 - e^{-\frac{(s_1 - s)^2}{2\Delta s^2}}\right) \qquad \text{if } s > s_1 \quad (12)$$

Here, s_1 is the position of the step along the arclength (with $s = 0$ at $R = R_i$ and $s = s_{tot}$ at $R = \epsilon$). We determined s_1 numerically to obtain that the surface area defined by $s < s_1$ is the tip surface S_{tip}.

1.5 Fitting of experimental data

The 2D experimental profiles derived from electron microscopy imaging [3] are available online. They needed to be projected to an axisymmetric profile, since the theory could only consider axisymmetric shapes. For a given profile, we find the two points A,B where the membrane intersects an horizontal axis, at each height from the base. We

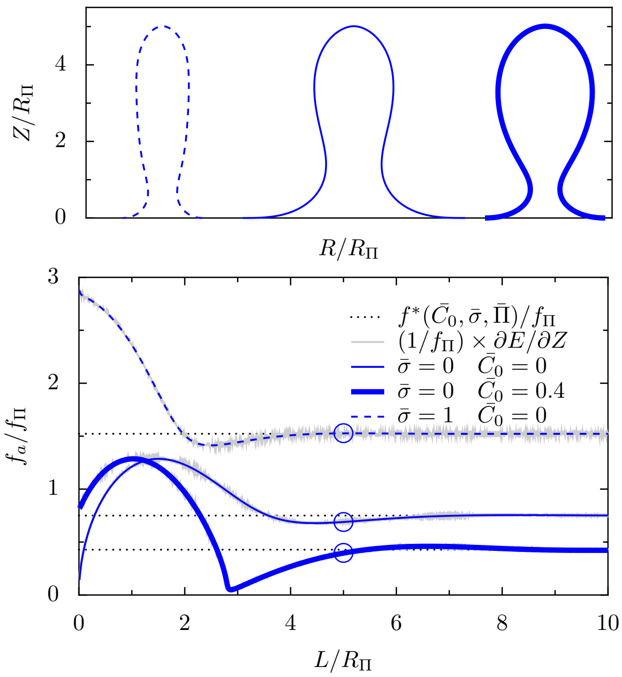

Figure 2. Top : three membrane shape profiles. Thin line: $\bar{C}_0 = 0, \bar{\sigma} = 0$, dashed line: $\bar{C}_0, \bar{\sigma} = 1.0$, and thick solid line: $\bar{C}_0, \bar{\sigma} = 0$. Bottom : force versus height of the invagination, for the three aforementioned parameter sets. The gray and noisy lines represent the numerical calculation of $\partial E/\partial L$, showing that our results are thermodynamically coherent. Dotted lines indicate the analytical predictions of the plateau force.

then extract the two angles of the membrane with respect to the vertical axis at points A and B, and we define the local orientation t as the average of these angles. The radius of an axisymmetric profile at this height is then $[AB]\cos(t)/2$.

In the fitting procedure, it is important to note that R_Π act as a scaling parameter, while $\bar{\sigma}$ and $\bar{C}_0$ control the shape of the invagination. The renormalized force f can be seen as a Lagrange multiplier controlling the height of the invagination. Therefore, we compared experimental shapes to theoretical profiles with a large range for $\bar{\sigma}$ and $\bar{C}_0$, and for each profile, we tried values of R_Π from $5nm$ to $35nm$. For a given set $\{\bar{C}_0, \bar{\sigma}, R_\Pi\}$, only one value of f_a produces the correct invagination height.

The error between a theoretical profile and an experimental shape was defined as :

$$\text{Error} = \sum_i \frac{(R_i^{th} - R_i^{exp})^2}{(R_i^{exp})^2} \tag{13}$$

Where the R_i are the radii of profile sections, for height between 0 and L, where L is the length of the invagination.

A range of parameters $\{\bar{C_0}, \bar{\sigma}, R_\Pi\}$, rather than a discrete set, can be used to fit each profile, while keeping the error below 5%. We found that most profiles could be fitted with $R_\Pi \sim 15-25nm$, $\bar{\sigma} \sim 0$, and $\bar{C_0} \sim 0$–0.5. The accuracy of the fit is undermined by the facts that we did not fit the original profiles but an axisymmetric projection. Moreover the solution space is degenerate, and bias are possible in the experimental methods.

2 Forces

2.1 Force as the derivative of the energy

In our physical theory, the force f_a pulling the invagination should satisfy the thermodynamic definition of a force, i.e. $f_a = -\partial E/\partial Z$, in which $E = \mathcal{F} + f_a L$ is the total internal energy. Hence f can also be seen as a Lagrange multiplier controlling the height of the invagination. We have several ways to test the validity of our force computation. First, we can compute analytically the force required to pull long invaginations ($L \gg R_\Pi$), that are mostly tubular [7]. In this configuration, the total energy of the system is dominated by the energy of the tubular portion, and the radius of the tube R^* can be found by minimizing the energy per unit length of tube:

$$E_{tube} = 2\pi R \left[\frac{\kappa}{2} \left(\frac{1}{R} - C_0 \right)^2 + \sigma \right] + \Pi\, \pi R^2 \tag{14}$$

$$\partial_R E_{tube} \Big|_{R^*} = 0\,. \tag{15}$$

The numerically computed plateau value for the force matches this analytic value $f^* = E_{tube}(R^*)$ perfectly (figure 2, bottom).

Second, to confirm that the force computation is exact for any L, we verified that the input value for f_a, found by the shooting method, corresponded to the thermodynamical definition of the force $\partial_L E$. The numerical estimation of $\partial_L E/f_\Pi$ is noisy (due to small error of on the height L and on the angle ψ at the tip), but indeed f always matches $\partial_L E/f_\Pi$ (figure 2, right, underlying grey lines).

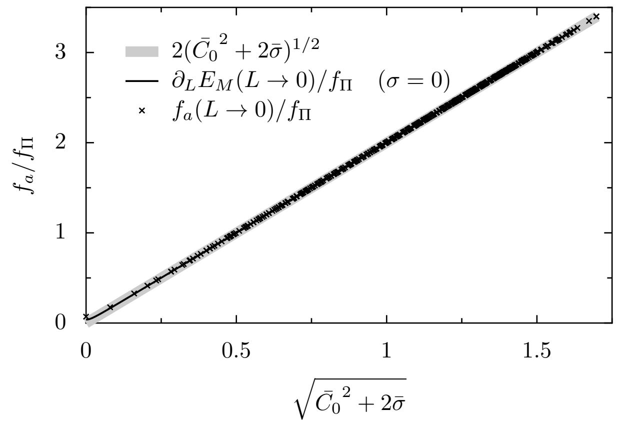

Figure 3. Initiation force f_0 as a function of physical parameters $\bar{C_0}$ and $\bar{\sigma}$. Black line : numerical solution in the Monge approximation. Crosses : numerical solution of the general shape equations. Grey line : fit by $f_0 = 2f_\Pi\sqrt{\bar{C_0}^2 + 2\bar{\sigma}}$.

2.2 Initiation force

At the early stages of endocytosis, before the appearance of a neck, we can describe the membrane in the Monge form using $Z(R)$ and $Z' = \partial Z/\partial R$ [8]. For early invaginations, we can further take the small deformation limit $Z' \to 0$, leading to a simpler formulation of the energy:

$$E_M \sim 2\pi\kappa \int_0^{R_i} \left[(\Delta Z - \bar{C_0})^2 2\bar{\sigma} Z'^2 + 2\bar{\Pi} Z\right] R dR \,. \tag{16}$$

Taking the limit $\bar{\sigma} \to 0$, equations 3,4 become, for an invagination of renormalized length $\bar{L}$:

$$Z(R) = -\frac{1}{128} R^2 (R^2 + 32a) + \bar{L} + b\, R^2 \log(R) \,. \tag{17}$$

The parameters $\{a, b, R_i\}$ should satisfy the boundary conditions :

$$Z(R_i) = Z'(R_i) = 0 \,, \tag{18}$$

$$Z''(R_i) = -\sqrt{\bar{C_0}^2 + 2\bar{\sigma}} \,. \tag{19}$$

For any given $\bar{L}$, we can then compute numerically $Z(R)$, from which we deduce $E_M(\bar{L})$. We can then compute the force $\partial E_M/\partial \bar{L}$ numerically. We could compare the

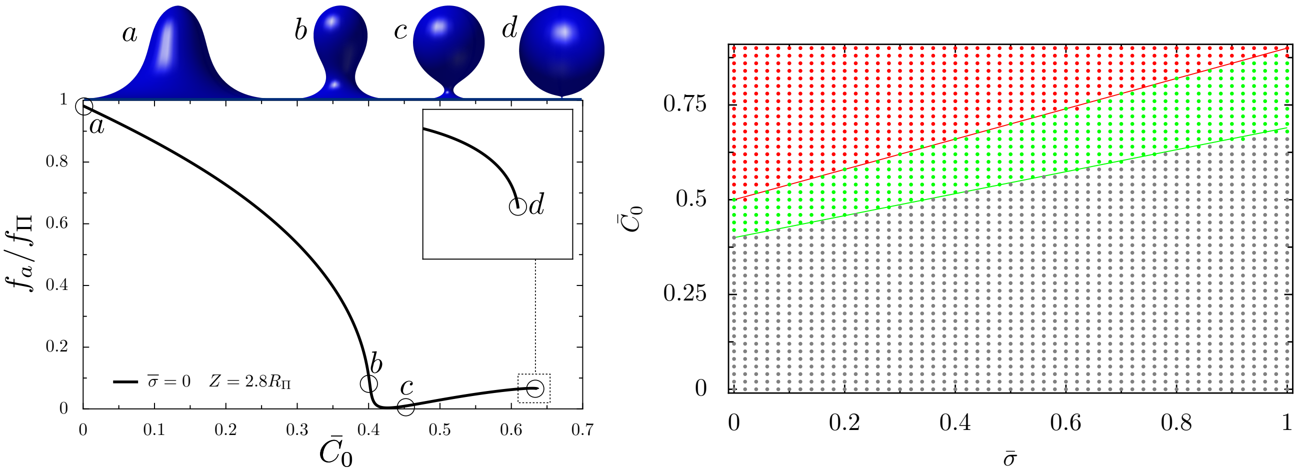

Figure 4. Left : Force as a function of spontaneous curvature for $L = 2.8R_\Pi$ and $\bar{\sigma} = 0$; the close-up shows that the branch ends at a finite $\bar{C}_0$. Right: Phase diagram of the budding instability as a function of spontaneous curvature $\bar{C}_0$ and tension $\bar{\sigma}$. Grey: no instability, green: instability with hysteresis, red: instability with discontinuity (lack of solutions for a range of L). Solid lines are visual guidelines showing that the critical value of $\bar{C}_0$ depends linearly on $\bar{\sigma}$.

approximate result from the Monge representation (in the absence of tension), the numerical result from the general membrane shape, and we find that they are well fitted by the formula :

$$f_0 = 2f_\Pi\sqrt{\bar{C}_0{}^2 + 2\bar{\sigma}} \tag{20}$$

3 Shape instability

3.1 Curvature-induced shape instability

It is interesting to compare the shape instability that we found with the pearling instabilities (see [9] and references therein), whereby (local) spontaneous curvature competes with membrane tension [10, 11], usually under constraints of volume conservation [9]. Comparing the energy of a tube (equation 2 with $\psi = \pi/2$ and $\dot{\psi} = 0$) to that of a string of spheres does not yield any instability in our case. Instead, upon increasing L at the transition, the bending energy decreases more than the pressure energy increases, and this transition occurs at heights where the membrane is far from tubular. This transition is therefore caused by the cost of bending the membrane at the tip and tail of the invagination, and these defects cost more energy in the quasi-tubular shape than in the spheroid shape, where the neck is a defect that can become infinitesimally small. The phase diagram (Fig. 4, right), shows that membrane tension

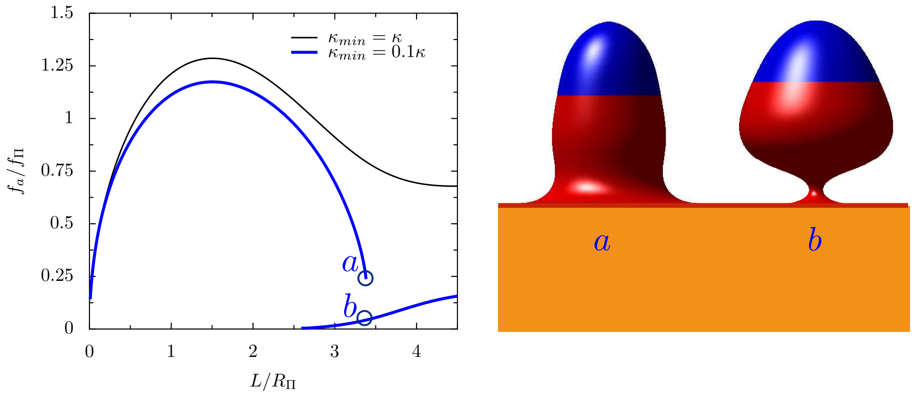

Figure 5. Left : pulling force (normalized by $f_\Pi = 2\pi\kappa/R_\Pi$) as a function of invagination length (normalized by R_Π). The blue line corresponds to a heterogeneous membrane (with a rigid tip of surface $S_{tip} = 2\pi R_\Pi^2$), while the black line shows the behavior for a homogeneous membrane, both in the absence of spontaneous curvature. Right : membrane profiles for the two solution branches. The blue surfaces indicate the rigid tips of the invaginations, while the red surfaces correspond to the more flexible regions.

actually inhibits this transition, which is due to membrane tubes of minimum energy having less membrane area than spheroids. The phase diagram also exhibit a region in which there is no stable membrane shape for a range of invagination heights, in contrast with the pearling instability. Despite the complexity of the equations, the phase boundaries exhibit a linear behavior for the critical value of $\bar{C}_0$ as a function of $\bar{\sigma}$ (Fig. 4, right).

3.2 Rigidity-induced shape instability

Strikingly, there is a shape instability even in the absence of spontaneous curvature if the membrane is heterogeneous, with a basal membrane of lesser rigidity $\kappa_{min} = \kappa\alpha_{min}$. This instability appears if κ_{min}/κ is smaller than a certain threshold, which depends on the surface area with higher rigidity. For example, with tip surface of $2\pi R_\Pi^2$, we found a threshold of $\kappa^*_{min} \sim 0.25\kappa$.

We then used $\kappa_{min} = 0.1\kappa$ (representative of the ratio of clathrin rigidity to membrane rigidity). Though the membrane shapes are greatly influenced by membrane heterogeneity (Fig. 5, right), their behavior as well as that of the force-distance curve (Fig. 5, left) is very similar to the curvature-induced instability, and it appears that both instabilities are of the same nature.

References

1. Jülicher F, Seifert U. Shape equations for axisymmetric vesicles: a clarification. Physical Review E. 1994;49(5):4728.
2. Dommersnes P, Fournier JB. N-body study of anisotropic membrane inclusions: Membrane mediated interactions and ordered aggregation. The European Physical Journal B-Condensed Matter and Complex Systems. 1999;12(1):9–12.
3. Kukulski W, Schorb M, Kaksonen M, Briggs JA. Plasma membrane reshaping during endocytosis is revealed by time-resolved electron tomography. Cell. 2012;150(3):508–520.
4. Berro J, Pollard TD. Local and global analysis of endocytic patch dynamics in fission yeast using a new ”temporal superresolution” realignment method. Molecular biology of the cell. 2014;25(22):3501–3514.
5. Lenz M, Crow DJ, Joanny JF. Membrane buckling induced by curved filaments. Physical review letters. 2009;103(3):038101.
6. Press WH. Numerical recipes 3rd edition: The art of scientific computing. Cambridge university press; 2007.
7. Derényi I, Jülicher F, Prost J. Formation and interaction of membrane tubes. Physical review letters. 2002;88(23):238101.
8. Nelson D, Peliti L. Fluctuations in membranes with crystalline and hexatic order. Journal de physique. 1987;48(7):1085–1092.
9. Tsafrir I, Sagi D, Arzi T, Guedeau-Boudeville MA, Frette V, Kandel D, et al. Pearling instabilities of membrane tubes with anchored polymers. Physical review letters. 2001;86(6):1138.
10. Bar-Ziv R, Moses E. Instability and pearling states produced in tubular membranes by competition of curvature and tension. Physical review letters. 1994;73(10):1392.
11. Derényi I, Koster G, Van Duijn M, Czövek A, Dogterom M, Prost J. Membrane nanotubes. 2007;p. 141–159.
